# Correction to: The CXCL12gamma chemokine immobilized by heparan sulfate on stromal niche cells controls adhesion and mediates drug resistance in multiple myeloma

**DOI:** 10.1186/s13045-021-01038-w

**Published:** 2021-02-16

**Authors:** Zemin Ren, Hildo Lantermans, Annemieke Kuil, Willem Kraan, Fernando Arenzana-Seisdedos, Marie José Kersten, Marcel Spaargaren, Steven T. Pals

**Affiliations:** 1 Department of Pathology, Amsterdam University Medical Centers, Loc. AMC, Meibergdreef 9, 1105 AZ Amsterdam, The Netherlands; 2 Lymphoma and Myeloma Center Amsterdam – LYMMCARE, and Cancer Center Amsterdam (CCA), Amsterdam, The Netherlands; 3 Department of Virology, Institut Pasteur, Paris, France; 4 Department of Hematology, Amsterdam UMC, University of Amsterdam, Amsterdam, The Netherlands

## Correction to: J Hematol Oncol (2021) 14(1):11 10.1186/s13045-021-01031-3

The original article [1] contained an error in the order of the citations in the manuscript which was mistakenly introduced by the production team of the journal and not by the authors of this article.

The article has since been corrected to ensure that all citations correspond to their appropriate references.
