# Supplementary material for: The CXCL12gamma chemokine immobilized by heparan sulfate on stromal niche cells controls adhesion and mediates drug resistance in multiple myeloma
Source: J Hematol Oncol. 2021 Jan 12;14:11. doi: 10.1186/s13045-021-01031-3 (PMC7802348; doi:10.1186/s13045-021-01031-3)

Supplemental material

Supplemental table 1: qPCR primer sequence

CXCL12α forward	CCAAACTGTGCCCTTCAGAT
CXCL12α reverse	CGTCTTTGCCCTTTCATCTC
CXCL12γ forward	CCAAACTGTGCCCTTCAGAT
CXCL12γ reverse	CTTTTCTGGGCAGCCTTTCT
RPLPO forward	GCTTCCTGGAGGGTGTCCGC
RPLPO reverse	TCCGTCTCCACAGACAAGGCCA

Supplemental figure legends

Figure 1. CXCL12α mRNA expression in HS5-WT and HS5-CXCL12γ KO cells. A representative for 2 independent experiments. ns, $P > 0.05$ using unpaired student's t-test.

Figure 2. MTT assay analysis of the growth of HS5-WT, HS5-CXCL12γKO and HS5-EXT1KO cells. Mean ±SD of three independent experiments in triplicate is shown.

Figure 3. (A) Adhesion of the HMCL L363 to HS5-WT and two HS5-CXCL12γKO clones. Mean ±SD of three independent experiments in triplicate. *, $P \leq 0.05$ using one-way ANOVA analysis. **(B)** Adhesion of the HMCL L363 to HS5-WT and HS5-EXT1KO. Mean ±SD of 3 independent experiments in triplicate. *, $P \leq 0.05$ using unpaired student's t-test.

Figure 4. (A) Flow cytometric discrimination of MM cells from BMSCs expressing GFP. **(B)** BMSC viability is not affected by bortezomib. HS5 cells were co-cultured with XG1 cells in the presence of bortezomib at indicated concentrations for 3 days. HS5 viability was analyzed by flow cytometry.

Figure 5. CXCL12γ mediates carfilzomib resistance in XG1 cells. XG1 was cultured alone, or co-cultured with HS5-WT, HS5-CXCL12γKO or HS5-EXT1KO, in the presence carfilzomib for 3 days. Mean ±SD of 3 independent experiments in triplicate. *, $P \leq 0.05$; **, $P \leq 0.01$ using one-way ANOVA analysis.

Figure 6. Recombinant CXCL12γ (or CXCL12α) induced adhesion to VCAM-1 does not protect HMCLs from bortezomib-induced cell death. The HMCLs XG1 or MM1.S were cultured on a surface co-coated with CXCL12γ or CXCL12α and VCAM-1 in the presence

of bortezomib for 3 days. Cell viability was analyzed by flow cytometry. A representative plot for 2 independent experiment is shown.

Supplemental data

Figure 1

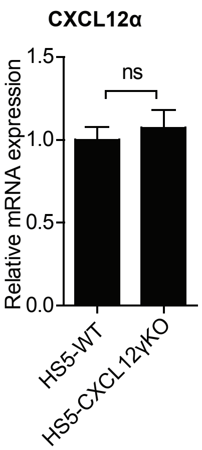

Figure 2

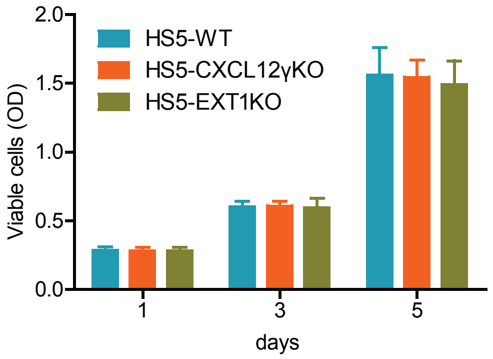

Figure 3

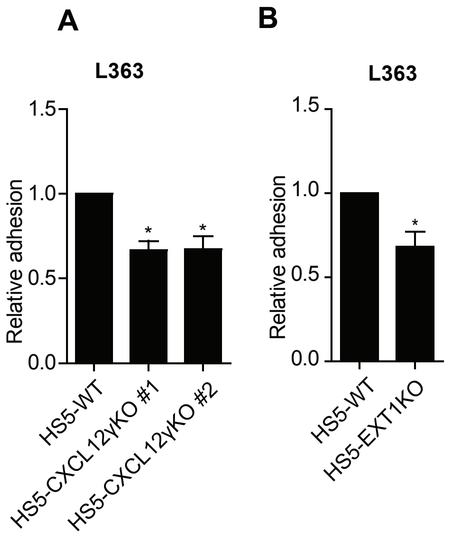

Figure 4

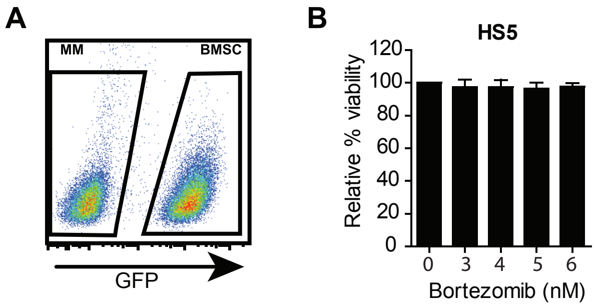

Figure 5

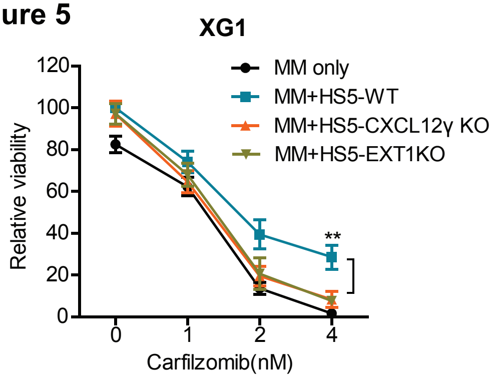

Figure 6

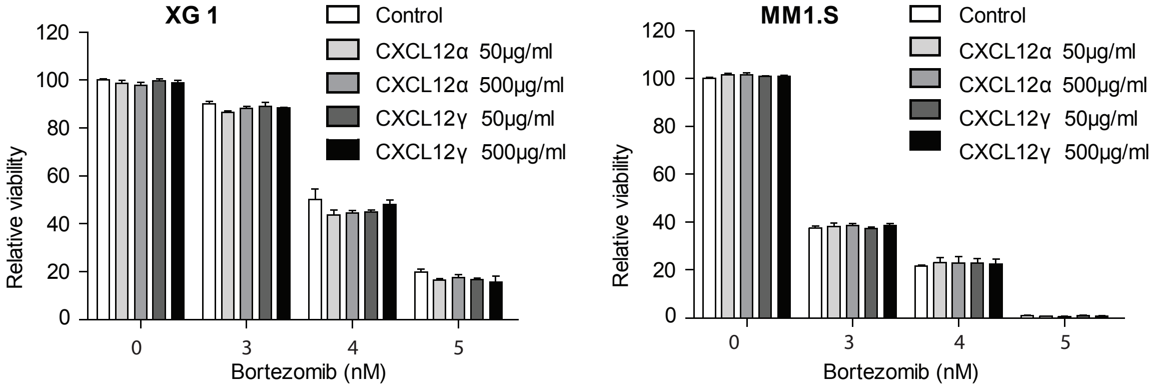

Supplement: Supplementary file 1 — Additional file 1. The CXCL12γ chemokine immobilized by heparan sulfate on stromal niche cells controls adhesion and mediates drug resistance in multiple myeloma. [file 13045_2021_1031_MOESM1_ESM.pdf]
